# Supplementary material for: Structural Characterization of Rat Galectin-5, an N-Tailed Monomeric Proto-Type-like Galectin
Source: Biomolecules. 2021 Dec 9;11(12):1854. doi: 10.3390/biom11121854 (PMC8699261; doi:10.3390/biom11121854)

Figure S1: Sedimentation velocity experiment of rGal5 in the absence (triangles) and in the presence (circles) of lactose.

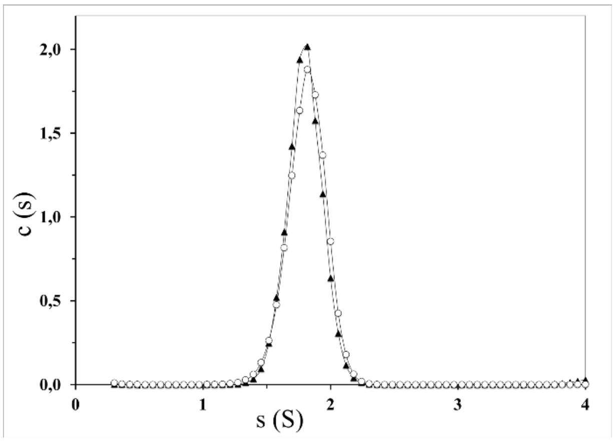

Figure S2: Binding of lactose, LacNAc and blood histo-group B (type 2) tetrasaccharide to rGal5 studied by Isothermal Titration Calorimetry.

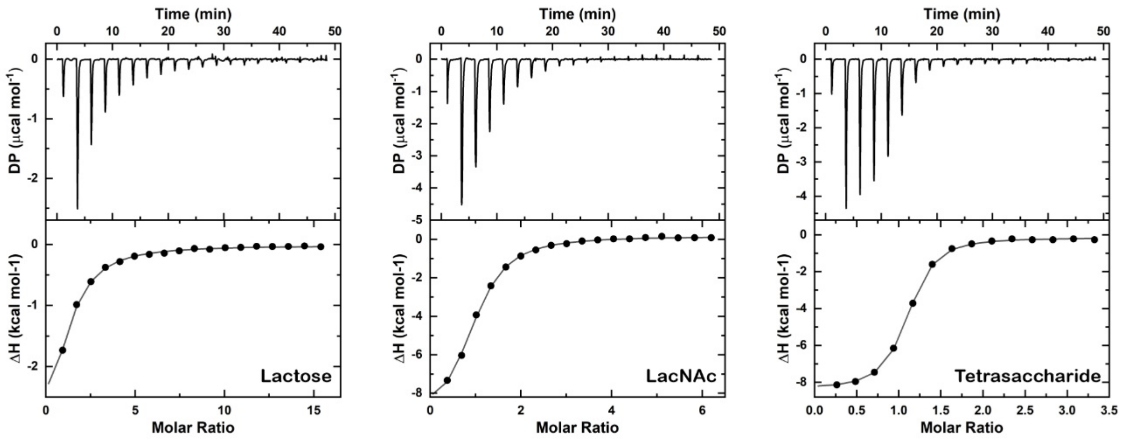

Supplement: Supplementary file 1 [file biomolecules-11-01854-s001.zip › rGal-5_Figures_Supplementary.pdf]
